# Timely Leukapheresis May Interfere with the “Fitness” of Lymphocytes Collected for CAR-T Treatment in High Risk DLBCL Patients

**DOI:** 10.3390/cancers14215276

**Published:** 2022-10-27

**Authors:** Mirko Farina, Marco Chiarini, Camillo Almici, Eugenia Accorsi Buttini, Francesco Zuccalà, Simone Piva, Irene Volonghi, Loris Poli, Simona Bernardi, Federica Colnaghi, Federica Re, Alessandro Leoni, Nicola Polverelli, Alessandro Turra, Enrico Morello, Anna Galvagni, Daniele Moratto, Duilio Brugnoni, Chiara Cattaneo, Emilio Ferrari, Andrea Bianchetti, Michele Malagola, Alessandro Re, Domenico Russo

**Affiliations:** 1 Unit of Blood Diseases and Bone Marrow Transplantation, Cell Therapies and Hematology Research Program, Department of Clinical and Experimental Sciences, University of Brescia, ASST Spedali Civili di Brescia, 25123 Brescia, Italy; 2 Diagnostics Department, Clinical Chemistry Laboratory, Flow Cytometry Section, ASST Spedali Civili di Brescia, 25123 Brescia, Italy; 3 Laboratory for Stem Cells Manipulation and Cryopreservation, Department of Transfusion Medicine, ASST Spedali Civili di Brescia, 25123 Brescia, Italy; 4 First Division of Anesthesiology and Critical Care Medicine, ASST Spedali Civili di Brescia, 25123 Brescia, Italy; 5 University Division of Anesthesiology and Critical Care Medicine, Department of Medical and Surgical Specialties, Radiological Sciences and Public Health, University of Brescia, ASST Spedali Civili di Brescia, 25123 Brescia, Italy; 6 U.O.C. Neurology Center for Neuromuscular Diseases, Department of Clinical and Experimental Sciences, University of Brescia, ASST Spedali Civili di Brescia, 25123 Brescia, Italy; 7 Research Center Ail (CREA), Chair of Hematology-Department of Clinical and Experimental Sciences, Unit of Blood Diseases and Stem Cell Transplantation, University of Brescia, ASST Spedali Civili di Brescia, 25123 Brescia, Italy; 8 U.O.C. Hematology, Department of Clinical Oncology, ASST Spedali Civili di Brescia, 25123 Brescia, Italy

**Keywords:** CAR-T, Lymphocytes fitness, pre-emptive lymphocytoapheresis, T-cells repertoire

## Abstract

**Simple Summary:**

Chimeric antigen receptor (CAR)-T cell therapy has revolutionized the treatment of specific hematological diseases, but is unsuccessful in around 60% of patients. The objective of the Bio-CAR-T BS study is to improve our understanding of the lymphocyte harvest to maximize the quality of the CAR-T cell product. We show here that “pre-emptive” lymphocyte apheresis (Ly-apheresis) of selected high-risk DLBCL patients may preserve the fitness of lymphocytes for CAR-T cell infusion. Specifically, the “pre-emptive” Ly-apheresis strategy resulted in a significantly higher CD4/CD8 ratio, significantly higher absolute counts and frequency of CD4+ naïve T cells, and a significantly higher frequency of CD8+ naïve T cells compared with the standard Ly-apheresis strategy (i.e., relapsed/refractory after two lines of treatments). Moreover, patients who underwent “pre-emptive” Ly-apheresis had a significantly lower frequency of CD8+ terminally differentiated T cells compared with standard Ly-apheresis.

**Abstract:**

The development of chimeric antigen receptor (CAR)-T cell therapy has revolutionized the treatment of hematological diseases. However, approximately 60% of patients relapse after CAR-T cell therapy, and no clear cause for this failure has been identified. The objective of the Bio-CAR-T BS study (ClinicalTrials.gov: NCT05366569) is to improve our understanding of the lymphocyte harvest to maximize the quality of the CAR-T cell product. Of the 14 patients enrolled, 11 were diagnosed with DLBCL, 2 with PMBCL, and 1 with ALL. Five of 11 DLBCL patients met the criteria for “pre-emptive” Lymphocytes-apheresis (being at high risk of second relapse), and 6 were included in the standard-of-care Lymphocytes-apheresis group. Previous autologous stem cell transplantation (ASCT) and age were significantly different between the two groups. At the time of Lymphocyte-apheresis, patients in the “pre-emptive” group had more “fit” lymphocytes (higher CD4+/CD8+ ratio; higher naïve T cells levels) compared with standard group, probably due to the impact of ASCT. At the same time, also being older than 60 years results in a more “exhausted” lymphocyte profile. Overall, “pre-emptive” Ly-apheresis in DLBCL patients at high risk of relapse appears to be feasible and may allow the timely collection of “fit” lymphocytes for CAR-T cell manufacturing.

## 1. Introduction

Chimeric antigen receptor (CAR)-T cell therapy is conceivably the most innovative therapeutic strategy developed to treat specific hematological diseases over the last 5 years [1]. Its use has revolutionized paradigms related to the conventional chemotherapeutic approach, opening new opportunities for patients with advanced phase disease (i.e., relapsed/refractory). As new CAR-T cells are developed to treat other hematological neoplastic diseases, the therapeutic strategies for many of these will need to be revised in the future [1,2].

Two CAR-T cell products, axicabtagene ciloleucel (Axi-cel) and tisagenlecleucel (Tisa-cel), are currently registered in Italy for the third-line treatment of resistant/relapsed patients with acute lymphoblastic leukemia (ALL), diffuse large B-cell lymphoma (DLBCL), or primary mediastinal B-cell lymphoma (PMBCL) [3,4]. Clinically, these products have similar efficacy and safety despite their distinct constructs and different manufacturing processes [3,4,5,6]. Indeed, approximately 40% of patients with DLBCL, PMBCL, or ALL, who are resistant or relapsed after two lines of treatment, can obtain a durable complete remission with Axi-cel or Tisa-cel [7,8,9]. These results, demonstrated in pivotal registrational trials and real-world studies, are striking considering the patients did not respond to prior treatment approaches [7,8,9]. However, at least 60% of patients do not benefit from CAR-T cell therapy, and no clear causes for this failure have been identified.

Many factors could negatively impact the efficacy of CAR-T cells, including disease type, the burden of disease, or the number or type of prior treatment lines [6,10]. Notably, CAR-T cells generated from T-lymphocytes (T-Ly) enriched for early lineage T cells have durable persistence and replicative potential both in animal and in vivo models, while CAR-T cells from “exhausted” T-Ly (e.g., Terminally differentiated T-Ly) have been related with poorer response [10,11,12]. In particular, CAR-T cells with higher frequency of Naïve and Stem Cell memory T cells, as well as with an higher CD4+/CD8+ ratio at time of leukapheresis, have been related with better antitumor activity and clinical responses in multiple myeloma (MM) and Chronic lymphoblastic leukemia (CLL) patients [10,12]. Therefore, the number and the “fitness” of lymphocytes collected by leukapheresis might play a crucial role in CAR-T cells efficacy [13,14]. 

The definition of lymphocytes’ “fitness” is not yet fully delineated, but it can be described as the ability of a T cells to generate an efficient immune response and durable protection against infection or neoplasia. It is generally related to high level of CD4+ and CD8+ early lineage T cells (i.e., Naïve and Stem cell memory T Ly), which have high self-renewal capacity and may mature in cells with effector functions [15]. Conversely, persistent antigen exposure due to infection or neoplasia, lead T-Ly to exhaustion, resulting in loss of effector function and replicative capacity. T-Ly exhaustion was first described in a mouse model of lymphocytic choroid meningitis virus (LCMV) infection [16]. Interestingly, also treatments, especially chemotherapy, may lead to T-cells exhaustion. Indeed, naïve T-Ly declined progressively after treatments [17,18].

In the Bio-CAR-T BS study, we planned to anticipate Ly-apheresis (i.e., “pre-emptive” Ly-apheresis) in DLBCL patients who were likely to be at higher risk of relapse or refractive to second-line treatment in order to optimize the collection of higher frequency of early lineage T lymphocyte (“fit” lymphocytes). 

In doing so, we aim to maximize the quality of the CAR-T cell product and, ideally, the efficacy of CAR-T cells, improving CAR-T cells expansion and effector functions, as well as patient outcomes. T cells fitness at the time of Ly-apheresis was evaluated comparing T-Ly subpopulations between patients who underwent a “pre-emptive” or a standard Ly-apheresis program, evaluating in this way the impact of previous treatment, especially Autologous Stem Cell Transplantation (ASCT) on T cells repertoire. 

## 2. Materials and Methods

### 2.1. Study Design

The Bio-CAR-T BS study is an observational, prospective study enrolling consecutive patients with relapsed/refractory DLBCL, PMBCL, or ALL referred to ASST Spedali Civili of Brescia and eligible to receive Axi-cel or Tisa-cel, as per clinical practice (ClinicalTrials.gov: NCT05366569). 

Patients with B cell-ALL (≤25 years), DLBCL (18–70 years), or PMBCL (18–70 years) who were relapsed/refractory after two lines of treatments were considered eligible for CAR-T cell therapy (Tisa-cel or Axi-cel), according to the AIFA (Italian Medicines Agency) criteria. Additional inclusion criteria included, adequate performance status (0 or 1) and organ function, no active or uncontrolled infections, no thrombo-embolisms within the last 6 months, the absence of clinically relevant co-morbidities (e.g., select cardiovascular, neurologic, or immune disorders with organ dysfunction or requiring immunosuppressive treatment in the last 24 months), and a life expectancy of at least 3 months.

According to the Bio-CAR-T BS study design, we aimed, whenever possible, to submit selected high-risk DLBCL patients (see following section) to an early or “pre-emptive” Ly-apheresis program, ideally before ASCT, in order to evaluate the “fitness” or “exhaustion” of these lymphocytes compared with lymphocytes collected as standard-of-care (i.e., relapsed/refractory after two lines of treatments). Even if T cell exhaustion definition is still a subject of debate, it is generally considered as cellular differentiation associated with progressive declines in T Ly effector function in the setting of chronic antigen exposure [19]. On the other hand, the fitness of lymphocytes may be described as the ability of T cells to generate an efficient immune response and to have a high self-renewal. Usually, early lineage T cells (i.e., naïve and stem cell memory) are considered “fit” lymphocyte, as well as low expression of exhaustion functional markers, such as TIM-3, LAG-3 and PD-1 [20] on T cells. 

The study was approved by the local medical research Ethics Committee and it was conducted according to the criteria set by the declaration of Helsinki. Each subject signed an informed consent before participating to the study.

Lymphocytes were collected with TERUMO-BCT Spectra Optia Cell Separator applying the continuous Mononuclear Cell Collection (cMNC) collection software. Apheresis were performed both through peripheral veins or central venous catheter at different blood flow depending on patient characteristics and body weight and using Anticoagulant Citrate Dextrose (ACD) with a standard ratio set to 1:12. All the products meet the following specification requirements: viable total nucleated counted cells ≥ 2 × 10^9^ and viable CD3+ T cells ≥ 1 × 10^9^. This target is achieved processing 2–3 total blood volume depending on CD3+ cells basal count.

Lymphocytes collected from patients eligible for standard Ly-apheresis were sent immediately for CAR-T cell manufacturing. For “pre-emptive” Ly-apheresis, lymphocytes were initially cryopreserved and sent for manufacturing following activation of the CAR-T cell program. After manufacture (21–30 days), the CAR-T cells were returned to our hospital and infused in eligible patients. 

Primary objectives of the study were to (1) evaluate the feasibility of a “pre-emptive” Ly-apheresis program in high risk of secondary relapse/refractory DLBCL patients; the (2) assessment of T-Ly subpopulations at the time of Ly-apheresis in order to evaluate the impact of previous treatment, especially ASCT, on T cells fitness, and if (3) “pre-emptive” program may allow to collect more “fit” lymphocytes. Secondary objectives of the study were the (1) monitoring of CAR-T cells expansion after infusion, (2) efficacy and outcome of CAR-T cell infusion in the pre-emptive and standard groups, and the (3) safety of CAR-T cells infusion. 

### 2.2. “Pre-Emptive” Ly-Apheresis Program

Since cryopreservation of lymphocytes collected by apheresis before CAR-T cell manufacturing is permitted for the Tisa-cel product, selected high-risk DLBCL patients were designated for “pre-emptive” Ly-apheresis, ideally before ASCT, to cryopreserve lymphocytes for CAR-T cell manufacturing once the AIFA eligibility criteria were satisfied.

The rationale behind the “pre-emptive” Ly-apheresis strategy was to preserve the fitness of lymphocytes for CAR-T cell infusion. High-risk DLBCL patients eligible for “pre-emptive” Ly-apheresis included patients with primary refractory DLBCL, Intermediate/High IPI score DLBCL or DE/DH DLBCL patients with MRD/PET positivity before ASCT, and Low/Inter-Low IPI score DLBCL patients who had relapsed within 1 year of the first-line treatment (Table 1).

When the “pre-emptive” strategy could not be pursued, Ly-apheresis and CAR-T cell manufacturing were scheduled as standard-of-care (i.e., relapsed/refractory after two lines of treatments).

The design of this program was based on our 10-years of treatment experience on >500 DLBCL patients [21]. According to the algorithm of response data from our Center, we considered that approximately 15 to 21 patients/year could be enrolled to Ly-apheresis (either before [“pre-emptive”] or after two lines of treatments) (Table 1). 

This study design, therefore, allowed a direct comparison of the “fitness” or “exhaustion” of lymphocytes depending on the collection strategy undertaken.

### 2.3. CAR-T Cell Treatment

Bridging therapy, when needed, was allowed (e.g., radiotherapy; salvage chemotherapy). Before CAR-T cell infusion, patients received one cycle of lymphodepleting chemotherapy (fludarabine 25 mg/m^2^ of body surface area and cyclophosphamide 250 mg/m^2^ of body surface area given daily for 3 consecutive days). 

During hospitalization, patients were checked for the onset of cytokine-release syndrome (CRS) and immune effector cell-associated neurotoxicity syndrome (ICANS) every 8 and 12 hours, respectively, if symptoms occurred. Patients were discharged between 15 and 20 days post-CAR-T cell infusion and followed as out-patients. 

CRS was graded according to the CAR-T-cell-therapy–associated TOXicity (CARTOX) criteria and ICANS was graded by the CAR-T-cell–related encephalopathy syndrome criteria, with severe CRS and ICANS defined as grade ≥ 3 [22]. Tumor response assessments were performed according to the Lugano 2014 criteria [23].

### 2.4. Flow Cytometry Ly “Fitness” Analysis

Flow cytometry analyses were performed on peripheral whole blood before apheresis and on its individual components after apheresis. Cell staining was performed directly on 100 μl of the samples, or on diluted samples where appropriate, following standard manufacturer protocols using specific mixtures of monoclonal antibodies. All stained samples were acquired on a Canto II (BD Bioscience) flow cytometer and analyzed using DIVA software version 8.0.2. 

A combination of monoclonal antibodies (MoAbs) directed against CD3, CD4, CD8, CD16, CD56, CD19, and CD45 were used coupled with the use of TruCount beads in order to determine absolute counts of lymphocyte subsets. In addition, a mix of MoAbs directed against CD45RA, CD45R0, CCR7, CD31, CD3, CD4, and CD8 were used to evaluate T-lymphocyte subsets. In particular, the following subsets were analyzed: CD4+CD45RA+CCR7+CD31+ recent thymic emigrants; CD4+ and CD8+ naive (CD45RA+CCR7+), central memory (CD45RA-CCR7+), effector memory (CD45RA-CCR7-) and terminally differentiated (CD45RA+CCR7-) cells. 

In addition, the Bio-CAR-T BS study includes monitoring of CAR-T cells expansion after infusion. In accordance with current protocols, the evaluation of CAR-T cells necessitates indirect staining, therefore patients’ cells were stained with a specific biotinylated CD19 CAR Detection Reagent followed by incubation with an anti-biotin PE conjugated MoAb. Live CD4+ and CD8+ CAR-T cells were identified after exclusion of apoptotic and dead cells, based on the use of a live/dead marker (i.e., 7′AAD). This was undertaken at the following time points: at the time of Ly-apheresis (on both peripheral blood and the leukapheresis product), post-CAR-T cell infusion on days 1, 3, 7, 10, 14, and 21, and then monthly until 1 year, or at any time in the case of relapse or the onset of CRS or ICANS. 

### 2.5. Statistical Analyses

All stained samples were acquired on a Canto II (BD Bioscience) flow cytometer and analyzed using DIVA software version 8.0.2. Categorical variables were summarized as number and percentage and compared using unpaired T-test or Chi-square test, as appropriate. Continuous variables were summarized as median and range and compared using Mann-Whitney U test. All *p* values ≤ 0.05 were considered statistically significant. Statistical analysis was performed with EZR version 1.54.

## 3. Results

**Figure 1:**
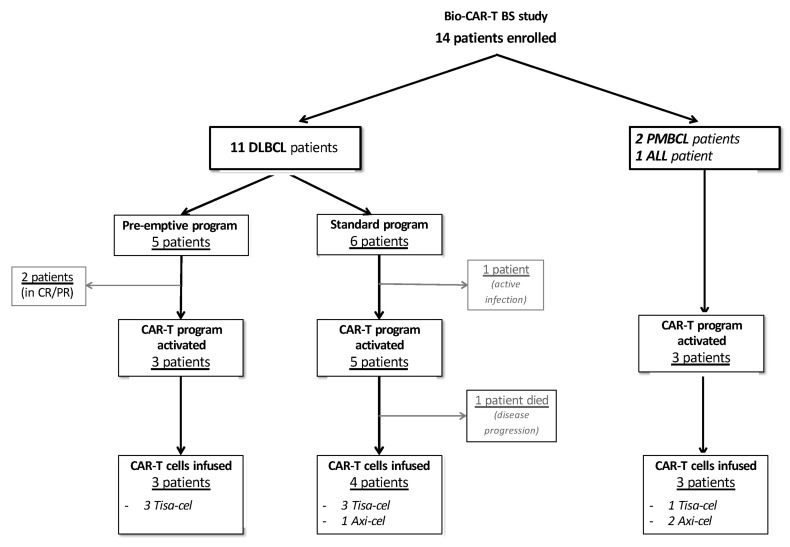
BIO-CAR-T BS study flow chart. ALL, acute lymphoblastic leukemia; CAR, chimeric antigen receptor; CR, complete remission; DLBCL, diffuse large B-cell lymphoma; PMBCL, primary mediastinal B-cell lymphoma; PR, partial remission.

A total of 14 patients were enrolled in the Bio-CAR-T BS study between April and January 2022. Patients were diagnosed with DLBCL (*n* = 11), PMBCL (*n* = 2), or ALL (*n* = 1) (Figure 1 and Supplementary Table S1).

Overall, 5 of 11 (45%) DLBCL patients met 1 of 3 criteria for the “pre-emptive” Ly-apheresis program (Table 1 and Supplementary Table S1). Of these 5 patients, 4 had PET positivity before ASCT, while 1 was primary refractory to first-line treatment. The clinical characteristics of patients with DLBCL in the “pre-emptive” and standard Ly-apheresis groups are summarized in Table 2. 

All patients in the standard group had undergone prior ASCT, while none in the “pre-emptive” group had (*p* = 0.002). Except for patient age, where the median age of patients in the “pre-emptive” group was significantly younger than in the standard group (51 versus 67 years, respectively; *p* = 0.02), no other between-group differences were identified at the time of Ly-apheresis (Table 2).

The CAR-T cell program was activated in 3 (60%) of 5 DLBCL patients in the “pre-emptive” group (all non-responsive to second-line treatment) and in 5 (83%) of 6 DLBCL patients in the standard group (non-responsive to second-line treatment *n* = 1; relapsed after two lines of treatment *n* = 4). Two patients in the “pre-emptive” group remain in complete remission after 11 months and 12 months, and 1 patient in the standard group was not eligible for the program because of an active infection (Figure 1, Table 2 and Supplementary Table S1). 

In patients who underwent “pre-emptive” Ly-apheresis and have activated a CAR-T program, the median number of days from leukapheresis to delivery of CAR-T cells was 89 days (range 55–98), while in the standard group this was 45.6 days (range 27–64) (*p* = 0.14) (Table 2).

Of the 5 DLBCL patients in the standard group who activated a CAR-T cell program, 1 died due to disease progression before receiving CAR-T cells, 3 were infused with Tisa-cel (median dose, 3.0 × 10^8^ CAR-positive viable T cells; range, 0.1–4.0 × 10^8^), and 1 with Axi-cel (2 × 10^6^ CAR-positive viable T cells/kg). All 3 patients from the “pre-emptive” group were infused with Tisa-cel (median dose, 3.0 × 10^8^ CAR-positive viable T cells; range, 2.0–4.0 × 10^8^) (Figure 1). Both PMBCL patients received Axi-cel (2 × 10^6^ CAR-positive viable T cells/kg for both), while the ALL patient received Tisa-cel (2.0 × 10^8^ CAR-positive viable T cells).

### 3.1. Lymphocyte Subpopulation Cell Analysis at Time of Ly-Apheresis

**Figure 2:**
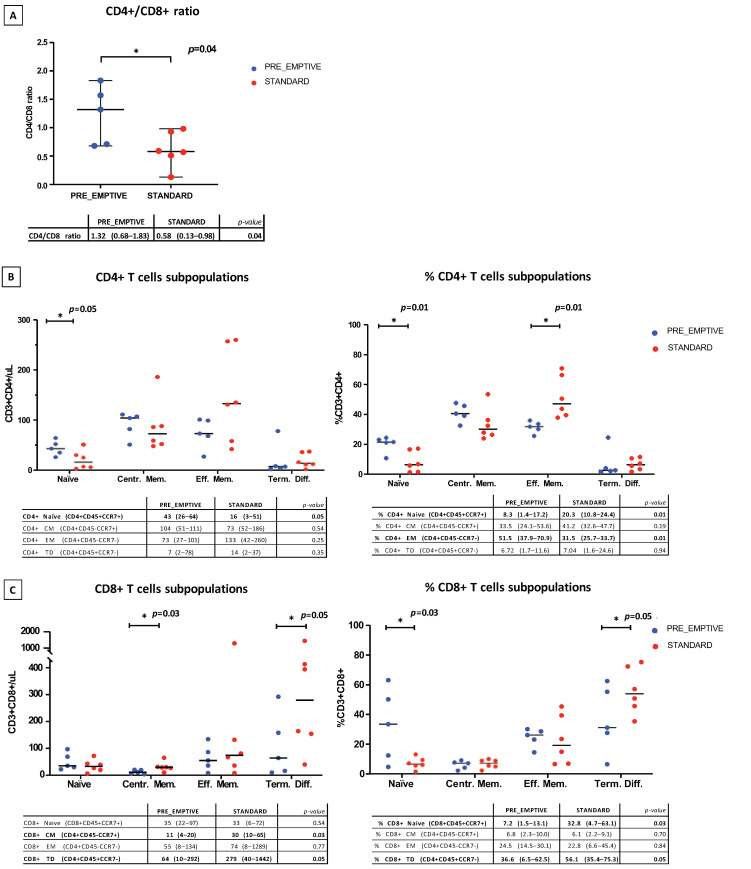
Ly-subsets in DLBCL patients who received “pre-emptive” (blue) or standard Ly-apheresis (red). (**A**) CD4+/CD8+ ratio. (**B**) CD4+ T cell subpopulations evaluated as absolute counts (cells/μL) and as a percentage. (**C**) CD8+ T cell subpopulations evaluated as absolute counts (cells/μL) and as a percentage. * = statistical significance (i.e., *p* < 0.05).

Lymphocyte subsets were analyzed before Ly-apheresis in DLBCL patients who underwent “pre-emptive” Ly-apheresis and in those who did not (standard group) (Figure 2). A significantly higher CD4+/CD8+ ratio was identified in patients in the “pre-emptive” Ly-apheresis strategy than those in the standard group (*p* = 0.04) (Figure 2A). Patients who underwent a “pre-emptive” Ly-apheresis also had a significantly higher absolute count of CD4+ naïve T cells (*p* = 0.05) (Figure 2B), a significantly higher percentage of CD4+ naïve T cells (*p =* 0.01) (Figure 2B) and of CD8+ naïve T cells (*p* = 0.03) (Figure 2C), a significantly lower percentage of CD4+ effector memory T cells (*p* = 0.01) (Figure 2B) and of CD8+ terminally differentiated T cells (*p* = 0.05) (Figure 2C), and significantly lower absolute counts of CD8+ central memory T cells (*p* = 0.03) and CD8+ terminally differentiated T cells (*p* = 0.05) (Figure 2C), compared with patients in the standard group. As all patients in the standard group had previously undergone ASCT, while none in the “pre-emptive” group had, the long-term effect of ASCT on the lymphocyte subpopulation could be assessed. This showed that the effect of ASCT on the lymphocyte subpopulation could be detected even after 1 year from the ASCT itself (results not shown).

**Figure 3:**
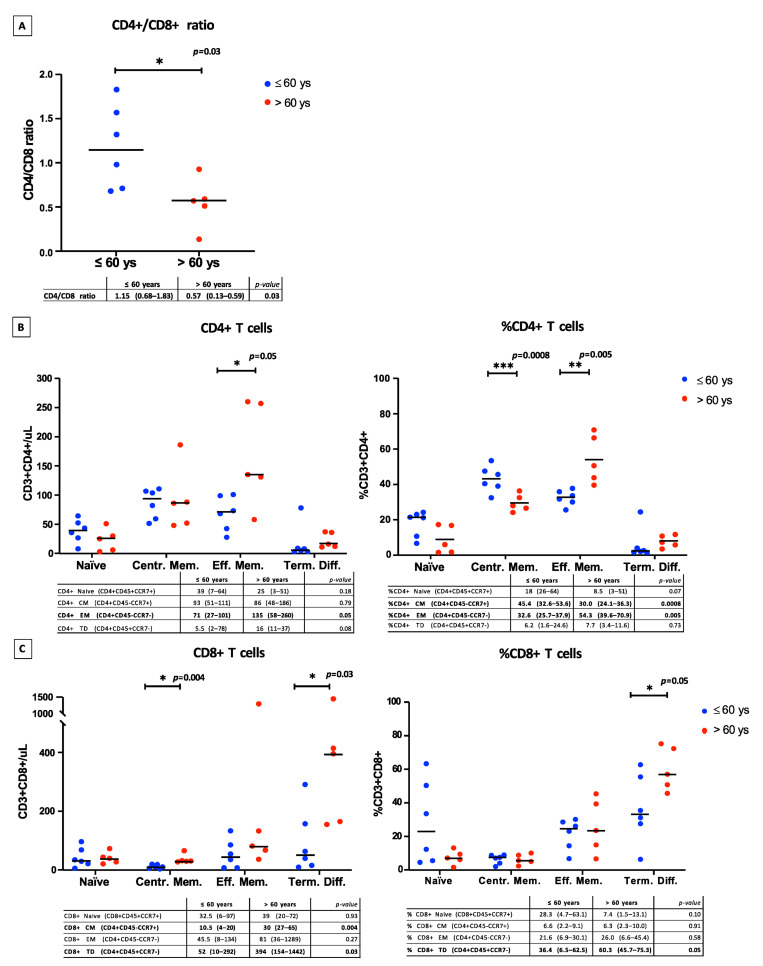
Ly-subsets in patients aged ≤60 years old (blue) and >60 years old (red). (**A**) CD4+/CD8+ ratio. (**B**) CD4+ T cell subpopulations evaluated as absolute counts (cells/μL) and as a percentage. (**C**) CD8+ T cell subpopulations evaluated as absolute counts (cells/μL) and as a percentage. * = statistical significance (i.e., *p* < 0.05); ** = statistical significance with *p*-value between 0.01 and 0.001. *** = statistical significance with *p*-value between 0.001 and 0.0001.

As age was significantly different between “pre-emptive” and standard groups, we evaluated its impact on the T cell repertoire. A significantly higher CD4+/CD8+ ratio was identified in patients aged ≤ 60 years compared with those aged > 60 years (*p* = 0.03) (Figure 3A). In addition, patients > 60 years had a significantly higher absolute count (*p* = 0.05) and percentage (*p* = 0.005) of CD4+ effector memory T cells, and a significantly lower percentage of CD4+ central memory T cells (*p* = 0.0008), compared with patients aged ≤ 60 years (Figure 3B). Moreover, patients aged > 60 years also had a significantly higher absolute count (*p* = 0.03) and percentage (*p* = 0.05) of CD8+ terminally differentiated T cells, as well as significantly higher numbers of CD8+ central memory T cells (*p* = 0.004) (Figure 3C), compared with patients aged ≤ 60 years. No other clinical or laboratory characteristics at time of Ly-apheresis impacted on T cell subpopulations. Patients with ALL or PMBCL had few circulating naïve T cells, and presented a lymphocyte repertoire that was similar to patients who underwent ASCT (i.e., DLBCL patients in the standard group).

### 3.2. Follow-Up Post CAR-T Cell Infusion

The median follow-up post infusion of CAR-T cells was 92 days (range 16–330). Six of 10 patients (60%) developed CRS after CAR-T cell infusion, while 1 patient developed ICANS in addition to CRS. Considering DLBCL patients in the pre-emptive group, 2 of the 3 obtained a partial remission by FDG PET at 1 month after CAR-T infusion, while one patient achieved complete remission. In the standard group, 3 patients had partial remission, while 2 obtained complete remission by FDG PET at 1 month after CAR-T infusion. One patient in the pre-emptive group relapsed 3 months after CAR-T cell infusion and died due to disease progression 1 month later. One patient in the standard group relapsed at 4 months after CAR-T infusion and he is now receiving salvage treatment. Finally, the ALL patient who received Axi-cel relapsed at 3 month after CAR-T infusion. All other patients are in complete remission or improved partial remission.

## 4. Discussion

With the introduction of CAR-T cell therapy, around 40–50% of eligible patients with relapsed/refractory DLBCL, PMBCL, or ALL can achieve a durable complete remission. However, the remaining 50–60% of patients still relapse and investigating the causes of CAR-T failure is very important. The number and the “fitness” of lymphocytes collected by leukapheresis can be some of the factors related to CAR-T cells inefficacy [13,14]. 

T cells collected by Ly-apheresis are phenotypically heterogeneous but are predominantly composed of effector memory T cells, while less than 10% are canonical central memory T cells [24,25]. Furthermore, some studies, both in vivo and in animal models, have shown that only central memory T cells, and other less differentiated T cell subsets such as naïve and the so-called “T-memory stem cells,” are critical for in vivo expansion, survival, and long-term persistence of CAR-T cells [11,26]. Naïve T cells and early lineage T cells, usually considered to be “fit” lymphocytes for their high self-renewal capacity, have lower expression of exhaustion functional markers, such as TIM-3, LAG-3 and PD-1 [20], and are correlated with higher in vivo and in vitro CAR-T expansion, as well as a better clinical outcome [27,28]. Conversely, CAR-T cells manufactured from “exhausted” T-lymphocytes have been related with poor clinical response [10,12]. 

In the Bio-CAR-T BS study, we planned to anticipate Ly-apheresis (i.e., “pre-emptive” Ly-apheresis) in DLBCL patients who were likely to be at higher risk of relapse or refractive to second-line treatment in order to optimize the collection of “fit” lymphocytes. 

It is known that naïve T cells decline progressively after therapy [17,18], and that when T cells are activated due to the persistent exposure to cancer antigen, they enter a state of exhaustion and fail to eradicate the neoplasia [29]. The optimal profile of T cell fitness is not well established to date. However, it can be described as the ability of a T cell to generate a CAR-mediated immune response resulting in the elimination of malignant cells and durable protection from disease relapse [15]. Notably, CAR-T cells generated from lymphocytes enriched for early lineage T cells have shown durable persistence and replicative potential in vivo [10,12]. 

Since the Tisa-cel manufacturing process allows cryopreservation of the leukapheresis product, early or “pre-emptive” Ly-apheresis could be utilized in suitable DLBCL or ALL patients. However, considering the low number of ALL patients potentially eligible for CAR-T cell treatment, our study was designed to undertake “pre-emptive” Ly-apheresis in patients with DLBCL only.

Based on our previous experience [21] and other studies [30,31], DLBCL patients considered at high risk of relapse before second-line salvage treatment were selected according to strict criteria (Table 1). Consequently, 5 of 11 DLBCL patients in this study were enrolled in a “pre-emptive” Ly-apheresis program, while 6 patients underwent Ly-apheresis following standard indications.

The “pre-emptive” Ly-apheresis strategy resulted in a significantly higher CD4/CD8 ratio, significantly higher absolute counts and frequency of CD4+ naïve T cells, as well as a significantly higher frequency of CD8+ naïve T cells compared with the standard Ly-apheresis strategy. Moreover, patients who underwent “pre-emptive” Ly-apheresis had a significantly lower frequency of CD8+ terminally differentiated T cells compared with standard Ly-apheresis. Therefore, the “pre-emptive” Ly-apheresis strategy may facilitate the collection of more “fit” lymphocytes. This result is consistent with data showing how conventional treatments may compromise the fitness of T cells for immunotherapies [32]. Moreover, our results are in line with a previous study in patients with multiple myeloma, which reported a leukapheresis product enriched in early lineage T cells and a higher CD4/CD8 ratio when lymphocytes were collected after response to induction therapy, while T lymphocytes collected from heavily relapsed/refractory patients showed a more “exhausted” phenotype [12]. Notably, an higher CD4+/CD8+ ratio at the of leukapheresis, as well as higher frequency of CD8+CD45RO-CD27+ T cells (i.e., early lineage CD8+ T cells) were the only factors associated with clinical response in these multiple myeloma patients. In addition, higher levels of naïve T cells in the leukapheresis product were also previously associated with higher CAR-T cell expansion and efficacy [11,26]. A recent review also emphasizes timely leukapheresis and cryopreservation as a safe and viable strategy [33]. After this preliminary analysis focused on T-cells repertoire before Ly-apheresis, correlations between the fitness of collected Ly and CAR-T cells expansion will be performed in the next future. Since all patients in the standard group were treated previously with ASCT, while none in the ”pre-emptive” group were, we considered that the difference in the T cell repertoire between the “pre-emptive” and standard groups could be due to ASCT, which results in a more “exhausted” lymphocyte profile before leukapheresis. Notably, the effect of ASCT on the lymphocyte subpopulation remained detectable at 1 year after ASCT.

We also found that patients aged >60 years had a significantly higher number and frequency of CD4+ effector memory T cells and CD8+ terminally differentiated T cells, but not CD4+ and CD8+ naïve T cells. Indeed, in our study, CD4+ and CD8+ naïve T cells were only influenced by previous treatment with ASCT. This result may be consistent with previous studies showing that increased age compromises T cell immunity [32,34].

Our study is limited by the low patient numbers, however, to our knowledge, these preliminary results are the first reported in this setting of patients according to a timely Ly-apheresis program. Even if a significative differences in frequency of early lineage T cells and exhausted T Ly have been described between pre-emptive and standard groups, further studies on larger populations are needed to validate these results. 

Despite the short follow-up, more than half of the patients in the “pre-emptive” group subsequently activated a CAR-T cell program, highlighting the feasibility of a “pre-emptive” Ly-apheresis strategy in clinical practice, selecting patients really at higher risk of relapse/refractory to the treatment, and its cost-effectiveness. Indeed, a pre-emptive Ly-apheresis program, on one hand, allow to collect more “fit” lymphocytes which may results in a better CAR-T efficacy and, therefore, in a reduced need for further treatments and their related direct and indirect (e.g., hospitalization) costs. On the other hand, having already cryopreserved lymphocytes at the time of relapse/refractory to the second line, they may be sent for manufacturing without wasting time and thus potentially reducing the duration of bridging therapy and avoiding further disease progression, which may eventually lead to patient’s death (e.g., in our study, one patient from the standard group died for disease progression before receiving CAR-T cells). 

## 5. Conclusions

In conclusion, “pre-emptive” Ly-apheresis in DLBCL patients at high risk of relapse appears to be feasible and may allow the timely collection of “fit” lymphocytes for CAR-T cell manufacturing. ASCT and older age (i.e., >60 years) may negatively impact the different subsets of T cells in DLBCL patients, resulting in a more “exhausted” lymphocyte profile. The Bio CAR-T BS study is ongoing, and other studies on larger populations are needed to confirm our preliminary results.

## Figures and Tables

**Table 1:** Pre-emptive Ly-apheresis program guidelines. ASCT, autologous stem cell transplantation; CAR, chimeric antigen receptor; DE, double-expresser; DH, double-hit; DLBCL, diffuse large B-cell lymphoma; IPI, International Prognostic Index; Ly, lymphocytes; MRD, minimal residual disease; N°, number; PET, positron emission tomography.

Type of Selection	Criteria for “Pre-Emptive” Ly-Apheresis Selection	N° Cases/Year Expected	N° Cases Observed ^1^	Ly-Apheresis Performed	CAR-T Program Activated	CAR-T Infusion Performed
A	Primary refractory DLBCL	6	1	1	1	1
B	Inter/High IPI score DLBCL or DE/DH DLBCL patients with MRD/PET positivity before ASCT	6	4	4	2	2
C	Low/Inter-Low IPI score DLBCL patients who had relapsed within 1-year of the first-line treatment	4	0	0	0	0

^1^ From April 2021 to January 2022.

**Table 2:** Clinical characteristics of patients with DLBCL in the “pre-emptive” and standard Ly-apheresis groups. ASCT, autologous stem cell transplantation; CAR, chimeric antigen receptor; DLBCL, diffuse large B-cell lymphoma; ECOG, Eastern Cooperative Oncology Group; IPI, International Prognostic Index; Ly, lymphocytes; *n*, number.

	Total	Pre-Emptive Ly-Apheresis	Standard Ly-Apheresis	*p*-Value
Number, *n* (%)	11	5 (45%)	6 (55%)	
Age, years, median (range)	60 (29–70)	51 (29–60)	67 (56–70)	**0.02**
Male, *n* (%)	3 (27%)	2 (40%)	1 (17%)	0.55
ECOG, *n* (%)				>0.99
0	5 (45%)	2 (40%)	3 (50%)	
1	6 (55%)	3 (60%)	3 (50%)	
IPI, *n* (%)				0.24
0–2	5 (45%)	1 (20%)	4 (67%)	
3–5	6 (55%)	4 (80%)	2 (33%)	
Ann Arbor score, *n* (%)				>0.99
0–II	1 (9%)	0 (0%)	1 (17%)	
III–IV	10 (91%)	5 (100%)	5 (83%)	
Disease state at Ly-apheresis, *n* (%)				
Primary refractory (A)	1	1 (20%)	*n*/a	
Partial response after 1 line (B)	4	4 (80%)	*n*/a	
Relapse after I line (C)	0	0	*n*/a	
Non-responsive > II line	3	*n*/a	3 (50%)	
Relapse after II line	3	*n*/a	3 (50%)	
Previous ASCT, *n* (%)	6 (43%)	0 (0%)	6 (100%)	**0.002**
CAR-T cell program activated, *n* (%)	8 (73%)	3 (60%)	5 (83%)	0.46
Tisa-cel	6	3	3	
Axi-cel	2	*n*/a	2	
State of disease at CAR-T cell program activation, *n* (%)				0.15
Non-responsive > II line	4	3 (100%)	1 (20%)	
Relapse after II line	4	0 (0%)	4 (80%)	
Leukapheresis to CAR-T cell delivery, days, median (range)	59 (27–98)	89 (55–98)	45.6 (27–64)	0.14

## Data Availability

For data sharing, contact the corresponding author: Deidentified individual participant data that underlie the reported results will be made available 3 months after publication for a period of 5 years from the corresponding author [MF] on request.

## Supplementary Materials

The following supporting information can be downloaded at: https://www.mdpi.com/article/10.3390/cancers14215276/s1, Table S1: Patient characteristics at the time of lymphocyte apheresis
