# Supplementary material for: Timely Leukapheresis May Interfere with the “Fitness” of Lymphocytes Collected for CAR-T Treatment in High Risk DLBCL Patients
Source: Cancers (Basel). 2022 Oct 27;14(21):5276. doi: 10.3390/cancers14215276 (PMC9655620; doi:10.3390/cancers14215276)
Supplement: Supplementary file 1 [file cancers-14-05276-s001.zip › cancers-1934651-supplementary.pdf]

Supplementary Material

Table S1. Patient characteristics at the time of lymphocyte apheresis

Pt	Sex	Age	Disease	IPI score	State of disease	Pre-emptive Ly-apheresis (category)	N° of lines before Ly-apheresis	Prior ASCT	Did patient receive CAR-T?	CAR-T cell
1	F	57	DLBCL	High	Relapse	No	2 (R-CHOPx6+ASCT)	Yes	Yes	Tisa-cel
2	F	51	DLBCL	Int/High	Partial response	Yes (B*)	1 (R-ESHAPx2)	No	No, in CR post II line	-
3	F	70	DH-DLBCL	High	Refractory	No	2 (R-DAEPOCHx6 +ASCT)	Yes	No, patient died	-
4	F	69	DE-DLBCL	Int/High	Partial response	No	2 (R-CHOPx6+ASCT)	Yes	Yes	Tisa-cel
5	F	61	DH-DLBCL	Int/Low	Partial response	No	2 (R-DAEPOCHx6 +ASCT)	Yes	No, active infection	-
6	M	59	DH-DLBCL	Int/High	Partial response	Yes (B*)	1 (R-DAEPOCHx6)	No	Yes	Tisa-cel
7	F	29	DE-DLBCL	Int/Low	Partial response	Yes (B*)	1 (R-DAEPOCHx6)	No	No, in CR post II line	-
8	F	47	DLBCL	Low	Refractory	Yes (A**)	1 (R-ESHAPx4)	No	Yes	Tisa-cel
9	M	25	B-cell ALL	NA	Refractory	No	2 (NILG protocol; blinatumomab)	No	Yes	Tisa-cel
10	F	50	PMBCL	NA	Relapse	No	2 (R-CHOPx6; Pembrolizumabx4)	No	Yes	Axi-cel
11	M	66	DH-DLBCL	Low	Relapse	No	2 (R-DAEPOCHx6 +ASCT)	Yes	Yes	Tisa-cel
12	F	69	DLBCL sec a FL	Int/Low	Relapse	No	3 (R-CHOPx6; R-ESHAPx3+ASCT)	Yes	Yes	Axi-cel
13	M	60	DE-DLBCL	Int/High	Partial response	Yes (B*)	1 (R-DAEPOCHx6)	No	Yes	Tisa-cel
14	F	18	PMBCL	NA	Refractory	No	4 (R-EPOCH; R-ICE; Nivolumab; Brentuximab)	No	Yes	Axi-cel

ALL, acute lymphoblastic leukemia; ASCT, autologous stem cell transplantation; CAR, chimeric antigen receptor; CR, complete remission; DE, double-expresser; DH, double-hit; DLBCL, diffuse large B-cell lymphoma; F, female; IPI, International Prognostic Index; Ly, lymphocytes; M, male; MRD, minimal residual disease; N°, number; NA, not applicable; PET, positron emission tomography; PMBCL, primary mediastinal B-cell lymphoma.

*B = Inter/High IPI score DLBCL or DE/DH DLBCL patients with MRD/PET positivity before ASCT

**A = Primary refractory DLBCL
